# Retraction Note: Circular RNA circ-CPA4/ let-7 miRNA/PD-L1 axis regulates cell growth, stemness, drug resistance and immune evasion in non-small cell lung cancer (NSCLC)

**DOI:** 10.1186/s13046-025-03499-0

**Published:** 2025-08-12

**Authors:** Weijun Hong, Min Xue, Jun Jiang, Yajuan Zhang, Xiwen Gao

**Affiliations:** 1 Department of Respiratory Medicine, Minhang Hospital, Fudan University, 170 Xin-Song Road, Shanghai, 201199 China; 2 State Key Laboratory of Genetic Engineering, Shanghai Engineering Research Center of Industrial Microorganisms, School of Life Sciences, Fudan University, Shanghai, 200438 China

**Retraction Note:** ***J Exp Clin Cancer Res*** **39, 149 (2020)**

10.1186/s13046-020-01648-1.

The Editor-in-Chief has retracted this article. After publication, a number of image concerns were raised, specifically:

- Figure 3 L was found to overlap with 3 C of a now retracted article, published previously [1].
- Blots from Fig. 5A were found to overlap with Fig. 1C of another article under consideration at the same time [2], and an article published previously [3] despite describing different conditions.
- Figure 5 H was found to overlap with Fig. 2C of [4].
- Two panels describing different conditions were found to overlap within Fig. 6C.
- Multiple panels within Fig. 7A were found to overlap.
- Figure 7 A was found to overlap substantially with Fig. 3E of another article under consideration at the same time [5].
- Supplementary Figure S1D was found to overlap with Fig. 4G from [2].

The Editor has lost confidence in the data and conclusions of this article.

The authors have not responded to correspondence from the publisher regarding this retraction.
